# Risk factors and patterns of recurrence after curative resection in Gastroesophageal Junction Adenocarcinoma

**DOI:** 10.12669/pjms.35.5.963

**Published:** 2019

**Authors:** Sadaf Batool, Misbah Khan, Sana Amir Akbar, Ijaz Ashraf

**Affiliations:** 1 Sadaf Batool, MRCS. FCPS Trainee, Shaukat Khanum Memorial Cancer Hospital and Research Centre, Lahore, Pakistan; 2 Misbah Khan, FCPS. Fellow European Board of Surgical Oncology, Fellow Surgical Oncology, Senior Instructor Surgical Oncology, Shaukat Khanum Memorial Cancer Hospital, MRCS. Shaukat Khanum Memorial Cancer Hospital and Research Centre, Lahore, Pakistan; 3 Sana Amir Akbar, FCPS Trainee, Shaukat Khanum Memorial Cancer Hospital and Research Centre, Lahore, Pakistan; 4 Ijaz Ashraf, MBBS. FCPS Trainee, Shaukat Khanum Memorial Cancer Hospital and Research Centre, Lahore, Pakistan

**Keywords:** Gastroesophageal junction cancer

## Abstract

**Objectives:**

We looked at risk factors and patterns of recurrence following surgical treatment of Gastro-Oesophageal Junction carcinoma (GOJC).

**Methods:**

Electronic medical records of patients with GOJC undergoing resection with curative intent between Jan 2009 and June 2017 at Shaukat Khanum Memorial Cancer Hospital were reviewed. GOJ cancer was classified as per Siewert classification. Clinical and operative details were studied and data was analysed using SPSS 20.

**Results:**

During the study period, we identified 78 patients with GOJ adenocarcinoma (38 patients with GOJ Type-I, 16 with Type-II tumors and 24 patients with GOJ Type-III tumors). Median age was 56 years ± 1.1. Male to female distribution was 72 versus 28%. Carbo-Pacli /5-FU based XRT verses Magic protocol (p<0.015) and advanced pathological T.-stage (p-value<0.032) were found to be statistically significant risk factors for recurrence. After a median follow up of 17.8 months+/- 1.5, 20 patients developed recurrence of which five had local recurrence, three had regional recurrence, eight had distant metastases and four had both local and distant metastases.

**Conclusion:**

The incidence of recurrence following curative resection of GOJC is 25%. Type of neoadjuvant treatment, waiting time for surgery and advanced T-stage are a risk factor for recurrence.

## INTRODUCTION

Esophageal and gastric cancers are among eight major cancers that account for 60% of the global cancer burden regarding incidence and mortality.^1^ Incidence of adenocarcinoma of gastroesophageal junction (GOJ) is rising specially in Western world despite decrease in esophageal squamous cell carcinoma and gastric adenocarcinoma. This increase is mainly due to increase in prevalence of obesity.^2^ This is also expected to happen in the Eastern countries in the near future due to Westernization.^3^

Siewert and Stein proposed a topographical classification of GOJ adenocarcinoma in 1996. According to this classification, there are three types of GOJ tumors depending upon the tumor location. GOJ Type-I tumor is located between 1 and 5 cm above the anatomic GOJ (Adenocarcinoma of distal esophagus), Type-II tumor is located is located within one cm above and 2 cm below the GOJ (true carcinoma of cardia) and Type-III tumor is located between 2 and 5 cm below the anatomic GOJ (sub cardia gastric cancer).^4^,^5^

These tumors are distinct from esophageal and gastric cancers because of their borderline anatomic location and consequently their tendency to spread towards abdominal or thoracic lymphatics. Therefore, these tumors are best managed with multimodality approach.^6^ Despite advances in multimodality therapy, disease free survival was 43.2 months (24.9–61.4) within two years after completing treatment with neo adjuvant chemo radiotherapy (weekly administration of five cycles of neoadjuvant chemo radiotherapy (intravenous carboplatin [AUC 2 mg/mL per min] and intravenous paclitaxel [50 mg/m^2^ of body-surface area] for 23 days with concurrent radiotherapy (41.4 Grey, given in 23 fractions of 1.8 Gee for five days per week) followed by surgery.^7^

Risk factors and recurrence patterns after neo adjuvant treatment followed by complete resection (R0) for GOJ adenocarcinomas are not well described in literature especially from this part of the world. An insight into the clinic pathological features predictive of recurrence and its patterns might help to predict effectiveness of therapy, guide treatment strategy and control relapse. Therefore, we present a retrospective review of patients diagnosed with GOJ adenocarcinoma and treated with neo adjuvant therapy followed by complete resection during 8-year study period, at a single cancer center in Pakistan. Objective of this study was to describe risk factors and recurrence patterns in these patients according to Siewert classification.

## METHODS

Data from all patients operated for carcinoma of gastro-esophageal junction (GOJ) between 2009 and 2017 in Shaukat Khanum Memorial Cancer Hospital and Research Centre was collected from prospectively maintained electronic medical record.

GOJ ca was classified as per Siewert classification of GOJ adenocarcinoma.^5^ Type-I is adenocarcinoma of the distal part of the esophagus (N=39). Type-II is adenocarcinoma of the real cardia (N=17) and Type-III is sub-cardinal gastric adenocarcinoma (N=26)

All adult patients (age above 18 years and up to 80 years) with histopathology proven gastro-esophageal adenocarcinoma who underwent curative surgery without metastatic disease were studied. Minimum follow-up requirement was one year and patients with either peril-operative death or a shorter follow up were excluded.

Between January 2009 and June 2017, 158 patients were operated for distal esophageal and proximal gastric adenocarcinoma. Among them 83 patients fall into GO Junction adenocarcinoma definition. Out of them three patients were lost to follow up and two patients had died at six months and were excluded from study. There were only two cases of initial EUS stage T2 with a node positive status, hence practically no cases of early disease are included in our study. Also, as per protocol no cases of metastatic disease were included.

All patients were diagnosed by esophagogastroscopy and biopsy. Staging was done by clinical examination, endoscopic ultrasound, staging CT chest, abdomen and pelvis, PET CT and staging laparoscopy with peritoneal washings/sampling where appropriate, for peritoneal and liver metastasis to ensure M0 disease.

After staging all patients were discussed in MDT. All patients received neo-adjuvant treatment either as induction plus concurrent chemotherapy (Paclitaxel and Carboplatin / 5-FU based) and external beam radiation (total dose of 41.4 Greys in 1.8-Greys fractions)^8^ for GOJ Type- I and II or perioperative chemo (ECX, ECF or DCX)^9^ for Type II and III.

Patients underwent surgery in the form of transhiatal esophagectomy/3 stage esophagectomy for GOJ Type-1 while partial or total gastrectomy or Ivor Lewis Esophagectomy for Type II and III GOJ adenoca.

Patients were followed until death or up to six years after surgery with last date of follow up been 1^st^ June 2017 with a median follow-up time of 14 months (IQR eight -24 months). They were seen with clinical examination on a regular basis for five years in the outpatient clinic (at three monthly intervals for the first year and at 6-month intervals for second year and yearly for the next three years thereafter). Six monthly CT scan was done during initial two years and yearly for last three years of follow up. Endoscopy was not done as a routine procedure unless indicated by patient significant symptoms.

Patients were analysed for difference in risk to recurrence and disease free survival. Risk factors (age, gender, BMI, comorbidities, staging, grading, location of tumor, neo-adjuvant treatment, duration between neoadjuvant treatment and surgery, type of surgery, pathological staging, and margins) were compared among groups with and without recurrence (Table-I). Patterns of recurrence were analyzed. Margins were considered positive if tumor was located at or within one mm of closest resection margin on final histopathology report.

**Table I:** Patient Demographic and tumor characteristics.

Variable	No Recurrence N (%) 58(74.4%)	Recurrence N (%) 20(25.6%)	Total N (%) 78(100)
***Gender***			
Male	41(70.7)	15(75.0)	56(71.8%)
Female	17(29.3)	5(25.0)	22(28.2)
***Comorbidity Index^10^***			
0-1	54(93.1)	19(95.0)	73(93.6)
>1	4(6.9)	1(5.0)	5(6.4)
***Pathological tumor grade***			
Well differentiated	8(13.8)	0(0.0)	8(10.3)
Moderately	33(56.9)	14(70.0)	47(60.3)
Poorly	17(29.3)	6(30.0)	23(29.5)
***Tumor Location***			
Siewert Type-1	26(44.8)	12(60.0)	38(48.7)
Siewert Type-2	15(25.9)	1(5.0)	16(20.5)
Siewert Type-3	17(29.3)	7(35.0)	24(30.8)
***adjuvant type***			
Carbo-Pacli /5-FU based XRT	16(27.6)	8(40.0)	24(30.8)
Magic protocol	42(72.4)	12(60.0)	54(69.2)
***Type of Surgery***			
Esophagectomy	30(51.7)	13(65.0)	43(55.1)
Total gastrectomy	23(39.7)	7(35.0)	30(38.5)
Partial Gastrectomy	5(8.6)	0(0.0)	5(6.4)
***Oncologic Resection margin***			
Negative	54(93.1)	15(75.0)	69(88.5)
Positive	4(6.9)	5(25.0)	9(11.5)
***Nodal Index***			
<0.1	39(67.2)	10(50.0)	49(62.8)
0.1-0.2	10(17.2)	3(15.0)	13(16.7)
>0.2	9(15.5)	7(35.0)	16(20.5)
Mean ± Standard Error of the mean			
Age(years)	56 ± 1.3	55.7 ± 2.1	55.9 ± 1.1
≤ 55yrs	27(46.6)	10(50)	37(47.4)
>55yrs	31(53.4)	10(50)	41(52.6)
BMI (Kg/m2)	23.2 ± 0.5	22.3 ± 1.2	23 ± 0.5
<22	24(41.4)	11(55.0)	35(44.9)
≥22	34(58.6)	9(45.0)	43(55.1)
Interval between Neo-adjuvant and Surgery	2.7 ± 0.2	4.7 ± 1.4	3.2 ± 0.4
Standard	56(96.6)	17(85.0)	73(93.6)
Delayed	2(3.4)	3(15.0)	5(6.4)
Total number of lymph nodes harvested	16.5 ± 1.2	17.5 ± 1.5	16.8 ± 0.97
Number of positive nodes	1.7 ± 0.5	3.2 ± 0.9	2.1 ± 0.5
Length Of Follow-up (in months)	17.5 ± 1.7	18.9 ± 2.9	17.8 ± 1.5

Recurrence was defined as presence of disease six months after curative surgery. Recurrence was divided into loco-regional (including nodal recurrence) or distant recurrence. It was confirmed either radiologically (CT or PET CT) for regional and distant disease or via endoscopy and biopsy for local recurrence.

Disease free interval was defined as interval between surgery and last follow up or recurrence. Waiting time for surgery of more than six weeks after completion of neoadjuvant chemotherapy and more than 12 weeks after chemo radiation was considered as delayed.

### Statistical Analysis

It is a retrospective analytic study design. All data were inserted and analyzed with the Statistical Software Package for the Social Sciences Windows version 20.0 (SPSS, Chicago, Illinois, USA). Pearson Chi- square and Fischer’s exact test for categorical variables and one-way Anova for continuous variables, along with binary logistic regression for establishing univariate associations of all prognostic factors for assessment of risk to recurrence. While multivariate nominal regression analysis was utilized for multivariate assessment of the same. Cox- regression analysis was used to assess disease free survival and impact of prognostic factors on this variable. For purpose of regression analysis continuous variables were dichotomized according to their mean values or split into sub-groups according to relative clinical significance. For all practical purposes a p-value of 0.05 or less was considered as level of significance.

## RESULTS

Detailed TNM stage distribution pre and post neo-adjuvant is shown in Table-II. Detailed univariate and multivariate analysis was done for risk factors influencing recurrence Table-III. Prolonged waiting time for surgery and type of adjuvant treatment i.e. chemo radiotherapy verses perioperative chemotherapy was found to be statistically significant (p <0.05). Overall survival of study population was 16.42 + 1.42 and mean time to recurrence was 12 +/-2.1 months.

**Table II:** Detailed stage distribution pre and post neo-adjuvant

Variable	No Recurrence N (%) 58(74.4%)	Recurrence N (%) 20(25.6%)	Total N(%) 78(100)
***Tumor initial T stage^11^***			
T1, T2, T3	45(77.6)	16(80.0)	61(78.2)
T4	13(22.4)	4(20.0)	17(21.8)
***Tumor initial N stage***			
N0	16(27.6)	7(35.0)	23(29.5)
N+ve	42(72.4)	13(65.0)	55(70.5)
***Pathological T stage***			
T0	10(17.2)	0(0.0)	10(12.8)
Tis, T1, T2	13(22.4)	3(15.0)	16(20.5)
T3,T4	35(60.3)	17(85.0)	52(66.7)
***Pathological N stage***			
N0	36(62.1)	11(55.0)	47(60.3)
N1	14(24.1)	2(10.0)	16(20.5)
N2	3(5.2)	3(15.0)	6(7.7)
N3	5(8.6)	4(20.0)	9(11.5)

T: tumor T stage, N: nodal stage according to AJCC 7th edition^12^

**Table III:** Uni-variate and multi-variate regression analysis for risk of Recurrence

	Uni-variate Analysis			Multi-variate Analysis		

Potential Prognostic factors	Odds Ratio	p-value	95% CI	Adjusted Odds Ratio	p-value	95% CI
Age< 55 years vs. ≥ 55 years	0.87	0.802	0.32-2.41	1.20	0.798	0.30-4.77
Gender males vs. Females	0.80	0.781	0.25-`2.56	1.16	0.852	0.24-5.70
BMI (Kg/m2) <22 vs. ≥22	0.58	0.293	0.21-1.61	0.41	0.249	0.09-1.88
Comorbidity Index^10^ 0-1 vs. >1	0.71	1.000	0.08-6.76	1.22	0.887	0.08-19.54
Pathological tumor grades well and Moderately differentiated vs: Poorly differentiated	1.50	0.355	0.63-3.57	3.49	0.078	0.87-13.95
Tumor initial T stage^11^ T1, T2, T3 vs. T4	0.87	0.822	0.25-3.04	0.32	0.201	0.06-1.83
Tumor initial N stage N0 vs. N+ve	0.71	0.576	0.24-2.01	1.15	0.859	0.26-5.13
Tumor Location Siewert type 1 vs. Siewert Type-2 Siewert Type-3	0.88	0.676	0.49-1.59	1.04	0.925	0.44-2.44
adjuvant type Carbo-Pacli /5-FU based XRT vs. Magic protocol	0.57	0.400	0.20-1.66	0.11	0.015	0.02-0.66
Type of Surgery Esophagectomy vs. Total gastrectomy Partial Gastrectomy	0.53	0.176	0.21-1.33	0.37	0.375	0.04-3.32
Waiting time for Surgery Standard vs. Delayed	4.77	0.111	0.73-30.93	28.75	0.016	2.19-377.99
Oncologic complete Resection vs. Margin positive	4.50	0.043	1.07-18.88	1.84	0.531	0.27-12.44
Nodal Index <0.1 vs. 0.1-0.2 >0.2	1.70	0.086	0.93-3.10	1.90	0.167	0.77-4.69
Pathological T stage T0, Tis, T1,T2 vs. T3,T4	3.31	0.038	1.07-10.25	5.03	0.032	1.15-21.88

Cox-regression analysis was done for Disease free survival and Time to Recurrence and important factors affecting survival were shown as Kaplan Meier curves Figure 2a, 2b, 2c, 2d, 2e, 2f. One-year Disease free survival(DFS)l was 86% while three year DFS was 62%. One year for standard waiting time was DFS 89% versus 0% for prolonged waiting time. One and three year DFS was 99% for patients who received neo-adjuvant radiation in addition to chemotherapy versus 95 % and 79% for patients who only received perioperative chemotherapy.

**Fig.1:**
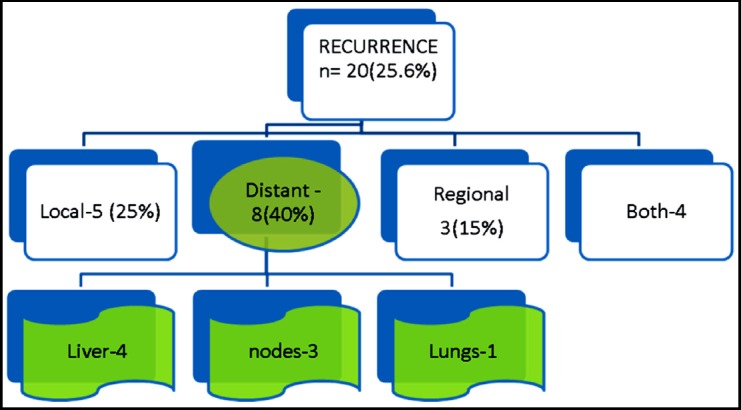


**Fig.2:**
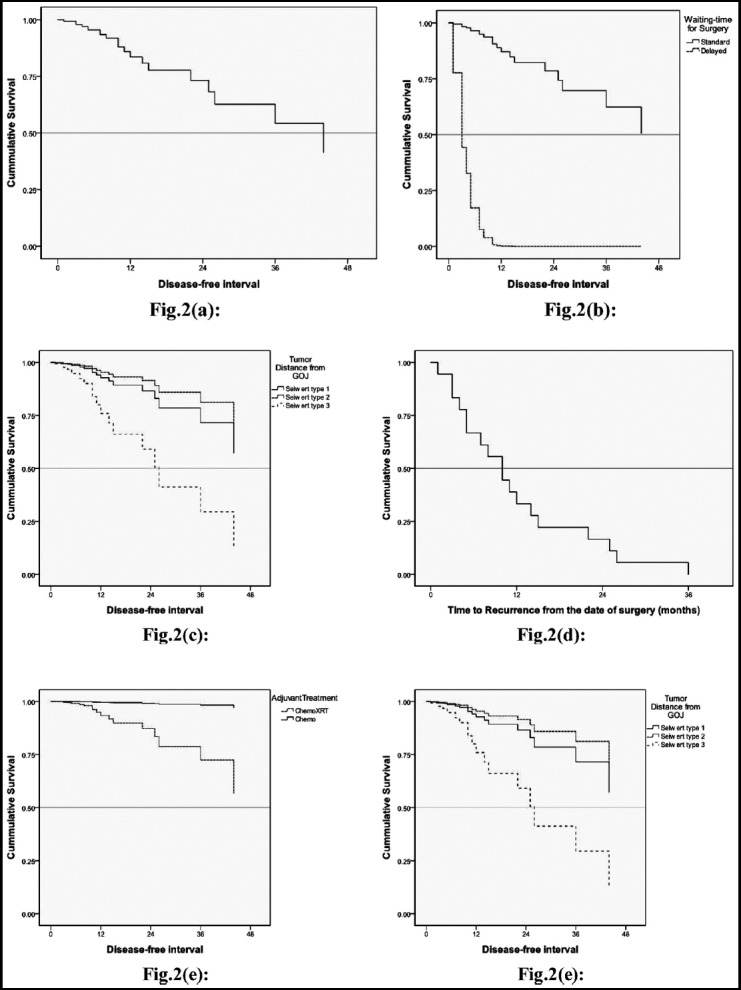


Similarly, the DFS for patients with node negative disease or a nodal index of <0.1 remained 99% at one and three years. In comparison, the one and three-year survival for a high nodal index was low (83 and 76%). Three year DFS was recorded for the group with nodal index 0.1-0.2 and >0.2 respectively and it was 50% and 49%, respectively.

DFS for Type-1 and Type-2 GOJ tumor at one year was similar at 94.5% and 96%, while Type-3 tumor had a low one-year survival of 76%. Three-year survival maintained similar pattern at one year for Type 1 and 2, 79% and 85% with Type-3 having 41%.

The majority of relapses occurred at distant sites and 61% recurrence occurred within the first year following surgery and 99.9 % of all at 26 months. The major site of distant recurrence was liver 45 % (n=nine) and lungs 15 %(n=three) (Fig.1).

## DISCUSSION

After 17.8 ± 1.5 months follow up, recurrence was seen in almost 25% of the study population despite R0 resection. Overall three-year survival rate was only 62%. It is of great concern as these patients were staged as having resectable localized disease. Moreover, outcomes of treatment for recurrent esophagogastric cancer is not encouraging.^12^

We have observed that preoperative staging process needs to be more accurate and extensive to correctly identify patients amenable to surgery as more than 60% of all recurrences developed within 12 months of surgery. High nodal index, prolonged waiting time for surgery after Neo-adjuvant therapy and type of neo-adjuvant therapy (perioperative chemo verse neo-adjuvant chemo XRT) were the risk factors for developing recurrent disease (*P* <0.05).

In order to minimize the incidence of recurrent disease in GOJ carcinoma, several strategies have been adopted. Most important of them was found to be early diagnosis. However, this (early T-stage pT0,1,2) could only be noticed in small number of study participants undergoing surgery (33.1%). Moreover. Early tumor extending only to submucosa can also spread to nodes^13^ or distant sites (15% verses 31). Incidence of distant and loco regional recurrence was similar in our study (40% each).

Extensive nodal harvesting was started such as 3 stage esophagectomy^14^ and radical lymphadenectomies^15^,^16^ assuming that it will improve survival by improving nodal staging and decreasing loco regional recurrence however it has not shown any survival advantage.^17^ Local recurrences could be minimized by surgery but distant recurrence needs to be managed by systemic therapies such as neoadjuvant/perioperative chemotherapy. Study conducted by Oppedijk V et al.^5^ has shown a 35% local recurrence rate following multimodal approach verses 58% in surgery alone group. We report a reduction in recurrence after neo-adjuvant chemo radiotherapy verses perioperative chemo (Magic trail) (p-value 0.01).

In spite of multimodal approach, local or regional recurrence and distant dissemination were the main cause of mortality in these patients so once identified through meticulous staging, high risk patients should be offered entry into trials of multimodality therapy or alternatively considered for palliative care

All patients with recurrence were referred to palliative care team. Patient with regional and distant recurrence were managed by palliative chemotherapy alone while patients with local recurrence were given symptomatic treatment such as stenting in addition to palliative chemotherapy.

### Limitations of the study

Firstly, relatively small group of patients were included in research over an extended time period with changing management options in terms of neo-adjuvant/adjuvant treatment modalities. Secondly, this study had a selection bias as only patients who underwent surgery after neo-adjuvant treatment were included, so patient who became irresectable or inoperable were not analyzed.
